# Knowledge of Mpox among secondary school students in Nigeria: a cross-sectional study

**DOI:** 10.11604/pamj.supp.2025.50.1.46070

**Published:** 2025-10-01

**Authors:** Agatha Nneka Obayi, Amelia Ngozi Odo, Lawreta Ijeoma Abugu, Cylia Nkechi Iweama, Olive Oluchi Ilo, Vivian Akah PaulUyonwu, Oliver Igwebuike Abbah

**Affiliations:** 1 University of Nigeria, Nsukka, Department of Human Kinetics and Health Education, Nsukka, Nigeria

**Keywords:** Mpox, knowledge, risk factors, secondary school students, preventive practices, socio-demographic factors, Nigeria

## Abstract

**Introduction:**

knowledge and control of Mpox are of great importance to public health in order to facilitate change in unhealthy behaviors that could predispose one to an infectious agent. The study aimed at determining the knowledge of Mpox among students in Enugu state, Nigeria.

**Methods:**

a cross-sectional survey research method was applied. A sample of 384 students, selected through random and purposive sampling methods, was used. A 27-item self-developed questionnaire was the data collection instrument designed to elicit information about students’ socio-demographics, knowledge about causes, transmission, signs and symptoms, and preventive practices of Mpox. Data was analysed using SPSS version 25, applying frequencies, percentages, means, standard deviation, and Chi-square statistic at a 0.05 level of significance.

**Results:**

only 41.3% had a good level of knowledge of Mpox disease. Secondary school students possessed an average level of knowledge of Mpox disease. 41% of students possessed a good level of knowledge of causes of Mpox; signs and symptoms (72.3%); transmission (40.7%), and prevention (65.7%). Age was significantly associated with a good level of knowledge of Mpox (p < 0.05), while gender, class level, and location were not significantly associated with a good level of knowledge of Mpox.

**Conclusion:**

students in secondary schools in Enugu state, Nigeria, had an average level of knowledge of Mpox disease. We recommend that Mpox education and awareness creation should go beyond school settings. The efforts of stakeholders during the COVID-19 outbreak should be replicated for the prevention and control of Mpox disease in Nigeria.

## Introduction

Monkeypox is a public health problem of great concern. It is caused by the Mpox virus (MPVX), a double-stranded DNA virus in the genus orthopoxvirus within the poxviridae family of viruses [1-3]. Initially, Mpox was believed to be endemic in West Africa only and in other African countries before 2022, but reports indicated cases of Mpox in other parts of the world [4-7]. This led to the WHO declaration of Mpox as a public health problem of international concern - PHEIC [8,9]. Globally, reports showed that in 2023, 87,858 cases and 143 deaths were reported from 111 countries between January 1 and May 30 [10]. America has 59,413 deaths (67.6%), Europe 25.02 (29.5%), Africa 17.94 (2%). The first human case was reported in 1970 in the Democratic Republic of Congo (DRC) [11], since then, a drastic increase was witnessed in these countries between 2000 and 2019 [12-15]. Furthermore, on 13th August 2024, the Africa Centre for Disease Control (CDC) declared Mpox resurgence cases in Africa a Public Health Emergency of Continental Security - PHECS [16]. This is because the Mpox can spread to other member states and needs a coordinated response.

Subsequently, Mpox outbreaks were reported in Nigeria, Sierra Leone, and Liberia, especially in children who had not received the vaccine for smallpox [8]. As of May 19, 2023, Nigeria has the highest reported cases of Mpox (842) in Africa, followed by DRC (739), Ghana (67), the Central African Republic (30), and Cameroon (29) [10]. The report also indicated that the period from September 2017 to August 2022 is the date for the re-emergence of Mpox in Nigeria, with 985 suspected cases, a case fatality ratio of Mpox in Nigeria was 3.0% with 985 suspected cases, 398 (40.4%) confirmed, and 12 deaths (3.0%) [17]. Since then, the outbreak has continued to rise. It was also observed that children less than 15 years of age accounted for 70 percent of cases to date in Africa during the ongoing 2024 outbreak. Studies have also linked infection to prolonged exposure and close contact with co-students and even school staff, as well as household members. Due to the nature of students´ involvement in sporting activities, learning, and recreational activities that involve body contact between and among them, which may pose a public health concern of infectious transmission, including Mpox, there is a need for the study. Man, as well as other primates, is a major transmitter of the virus, though the virus was first identified in monkeys [11,18].

Rodents, squirrels, rats, and dormice are suggested to be natural hosts of the virus [12,19]; thus, Mpox is transmitted from animals to humans through direct contact with the blood, body fluids, or cutaneous or muscular lesions of infected animals [19,20]. Human-to-human transmission has been reported through direct contact with infected materials from skin lesions of infected persons, through respiratory droplets following prolonged face-to-face contact, and contaminated objects such as cloths and beddings have been reported [6,19]. Transmission could also be through the placenta and childbirth [21,22], and the sexual route [8-10,21-25].

Symptoms of Mpox include fever, headache, muscle pains, general body weakness, and lymphadenopathy, which are the first phase. The second phase includes rashes for 1-3 days after fever onset. Also, Mpox asymptomatic cases have emerged recently following reports [25]. Lack of awareness of health workers, poor surveillance, weak health systems, inadequate number of laboratories, lack of access to approved antiretroviral medicine and vaccines, and stigmatization may result in under-reporting in Nigeria [13,26-28]. The report also showed that Nigeria, with a population of a median age of 18.6 years, most of whom have never been vaccinated against smallpox, has the largest pool of individuals susceptible to Mpox in the world [29]. Vulnerable individuals, such as women and children, are involved [30]. Monkeypox endemic countries also do not have adequate control infrastructure for prevention, detection, and treatment [31], which indicates that reports are lacking in so many schools and communities, thus, the need for the present study.

A strategy to interrupt local transmission of Mpox is urgently needed because its increase may have implications for global health. As more and more cases of Mpox are unfolding, there is a need for all hands to be on deck to contain this threat. Monkeypox knowledge exposure among the populations will benefit the schools, individuals, and communities, and will also add to the existing literature on Mpox through Mpox education and awareness. We therefore investigated knowledge of Mpox among secondary school students and also compared the knowledge level according to sociodemographic characteristics of the students.

## Methods

**Study design and setting:** we used the cross-sectional design to ascertain the knowledge of Mpox among secondary school students in Enugu State, Nigeria. Enugu state is in the southeast geopolitical zone and the capital of the old Eastern Region of Nigeria. It is also regarded as the coal city state due to coal mining activities of the colonial masters. The state is made up of 17 local government areas (LGAs) with three senatorial districts. Secondary schools in the state are managed by the Post-Primary Schools Management Board (PPSMB). For administrative purposes, each senatorial district is divided into education zones. In all, there are nine education zones, three in each senatorial district. The education zones serve as the administrative unit of the PPSMB.

**Population and sample:** the population of the study comprised all 9,794 secondary school students in the Nsukka education zone of Enugu state. A sample size of 384 determined through the Taro Yamane formula was used. Where sample size:


$$n=\frac{N}{1+N{\left(e\right)}^{2}}$$

Where, N = population; and e = 0.05 level of significance. A multi-stage sampling technique was used to select 384 students for the study. In the first stage, simple random sampling was used to select two secondary schools, one from rural and one from urban locations. In the second stage, 192 students were selected from each school through a random sampling method, 96 each from junior and senior secondary sections. Finally, purposive sampling was used to arrive at the number from each of the sections, making a total of 384 students selected for the study. Those who were in school were used.

**Data collection:** data for the study were collected using a researcher-designed structured questionnaire developed through a literature review. The questionnaire consisted of 27 items questions framed to elicit information about the students´ demographic information on age, gender, class level, location, and knowledge of Mpox. We asked students about their knowledge based on four knowledge categories: causes/predisposing factors; signs and symptoms; transmission and preventive practices of Mpox. Five questions were asked about causes/predisposing factors, five questions on signs and symptoms of Mpox, seven questions about mode of transmission, and six questions about prevention. The questionnaire was validated by experts in health education. Data collection was done during the students’ break period after obtaining permission from the school principals and teachers, who gave us access to the students. Data collection was done by the researchers after explaining the aim of the study and the procedure for filling out the questionnaire to the students. The filled-out questionnaires were collected on the spot. All data were collected within the first week of November 2024.

**Statistical analysis:** the questionnaire was sorted for completeness, and SPSS version 25 was used for data analysis. Only 300 filled-out questionnaires out of the 384 copies returned were used for data analysis. Eighty four (84) copies were discarded due to a lack of complete information either in the sociodemographic characteristics or in other sections of the questionnaire. The total score for questionnaire items was 100% for each of the knowledge categories and also for the overall knowledge. Furthermore, Bloom´s criteria [32] were used to assess the level of knowledge; thus, a score of less than 60% was regarded as a poor level of knowledge, 60% to 79% was considered an average level of knowledge, while a score of 80% and above was considered a good level of knowledge. Frequency counts and percentages were used to analyse the categorical variables, while mean and standard deviation were used to analyse the continuous variables. Significant differences were examined using the Chi-square test. The probability values less than 0.05 (p < 0.05) were considered significant.

**Ethical considerations:** ethical approvals for the study were granted by the Research Ethics Committee, Faculty of Education, University of Nigeria (REC/FE/2024/00030).

## Results

**Sociodemographic characteristics of study participants:** results show that most of the students studied are aged 15-19 years (61.0%), and were female (54.3%). More than one-half of the students (56.7%) are in senior secondary school, while almost one-third (60.3%) are in urban locations (Table 1).

**Table 1:** sociodemographic characteristics of study participants recruited from secondary schools in Enugu State, Nigeria, in November 2024 (N=300)

Characteristics	Frequency (F)	Percentages (%)
**Age (in years)**		
10-14	117	39.0
15-19	183	61.0
**Gender**		
Male	117	39.0
Female	163	54.3
**Class level**		
JSS 1-3	130	43.3
SSS1-3	170	56.7
**Location**		
Urban	181	60.3
Rural	119	39.7

Key: JSS= junior secondary school; SSS= senior secondary school

**Knowledge of Mpox among participants:** most students (93.0%) know that Mpox is transmitted through contact with body fluids, that fever is associated with Mpox (88.3%), that skin rash is a sign of Mpox (87.3%), and that Mpox can be transmitted by monkey (87.0%). However, less than one-half (40.0%) of participants know that Mpox can be transmitted from human to human. A little above one-half of students know that Mpox is caused by a virus (50.3%) and can be transmitted through blood transfusion (54.7%) (Table 2). On the level of knowledge of different categories of Mpox, 41% of students possessed a good level of knowledge of causes of Mpox with a mean knowledge score of 63.80 and a standard deviation (SD) of 24.758, while 72.3% possessed a good level of knowledge of signs and symptoms of Mpox with a mean score of 78.8 (SD = 26.195).

Furthermore, only 40.7% had a good level of knowledge of transmission (x̄ = 72.19; SD= 21.751) while 65.7% had a good level of knowledge of prevention (x̄ = 77.67; SD= 22.836). Overall, only 41.3% had a good level of knowledge of Mpox disease (x̄ = 73.23; SD 16.260). It can therefore be deduced that students had an average level of knowledge of Mpox disease, with the overall mean score of 73.23% (Table 3).

**Table 2:** knowledge of Mpox among study participants recruited from secondary schools in Enugu State, Nigeria, in November 2024 (N=300)

S/N	Items	Correct knowledge		Incorrect knowledge	
		F	%	F	%
1	Mpox is a zoonotic (animal) disease	183	61	117	39
2	Mpox is caused by a virus	151	50.3	149	49.2
3	Mpox is easily transmitted from human to human	120	40.0	180	60.0
4	Mpox is transmitted by monkeys and other wild animals to humans	261	87.0	39	13.0
5	Mpox is transmitted through the blood products of infected persons/animals	242	80.7	58	19.3
6	Skin rashes are one of the signs/symptoms of Mpox	262	87.3	38	12.7
7	Fever is associated with Mpox	265	88.3	36	11.7
8	Headache is a symptom of Mpox	253	84.3	47	15.7
9	Muscle pain is a sign/symptom	169	56.3	131	43.7
10	General body weakness is one of the signs/symptoms	233	77.7	67	22.3
11	Mpox is transmitted through contact with the body fluids of contaminated persons	279	93.0	21	7.0
12	Mpox can be contracted through the use of objects like clothes and bedding used by infected persons	196	65.3	104	34.7
13	Mpox is contracted through lesions (wound openings) on the skin	237	79.0	63	21.0
14	Mpox is transmitted through travel to an infected area	170	56.7	130	43.3
15	Mpox can be transmitted while preparing/eating meat from an infected animal	243	81.0	57	19.0
16	Mpox can be transmitted through respiratory droplets	227	75.7	73	24.3
17	Mpox can be transmitted through blood transfusion, placenta, and during childbirth	164	54.7	136	45.4
18	Wearing face masks prevents Mpox	207	69.0	93	31.0
19	Maintaining hand hygiene, that is, washing your hands at all times	265	88.3	35	11.7
20	Using hand gloves prevents Mpox	210	70.0	90	30.0
21	Always avoiding touching the eyes, nose/mouth with unwashed hands can prevent Mpox	235	78.3	65	21.7
22	Avoid eating or preparing infected meat/meat products	252	84.0	48	16.0
23	Avoiding unprotected sexual intercourse prevents Mpox	229	76.3	71	23.7

F = frequencies; % = percentages

**Table 3:** level of knowledge of Mpox among secondary school students in Enugu State, Nigeria, in November 2024 (N=300)

	Knowledge categories	Poor level; F (%)	Average level; F (%)	Good level; F (%)	x̄ (SD)
1	Causes	119 (39.7)	58 (19.3)	123 (41.0)	63.80 (24.758)
2	Signs and symptoms	46 (15.3)	37 (12.3)	217 (72.3)	78.80 (26.195)
3	Transmissions	92 (30.7)	86 (28.7)	122 (40.7)	72.19 (21.751)
4	Prevention	59 (19.7)	44 (14.7)	197 (65.7)	77.67 (22.836)
	Overall	55 (18.3)	121 (40.3)	124 (41.3)	73.23 (16.260)

F = frequencies; x̄ = mean; SD = standard deviation

**Associations between a good level of knowledge of Mpox disease and sociodemographic characteristics of participants:** overall, good knowledge of Mpox disease was higher among students aged 15-19 years (41.5%); males (41.6%); senior secondary students (41.8%), and those in urban locations (42.5%). Only age (χ2 =6.365; p= 0.041) was significantly associated with a good level of knowledge of Mpox disease. Gender, class level, and location were not significantly associated with a good level of knowledge of Mpox (p > 0.05) (Table 4)

**Table 4:** associations between a good level of knowledge of different categories of Mpox with socio-demographic characteristics of secondary school students in Enugu State, Nigeria, in November 2024 (N=300)

Characteristic	Knowledge of causes; F (%)	Knowledge of signs and symptoms; F (%)	Knowledge of transmission; F (%)	Knowledge of prevention; F (%)	Overall Knowledge; F (%)	Chi-square(χ^2^)	P-value
**Age (years)**							
10-14 (n=117)	57 (48.7)	90 (76.9)	40 (34.2)	83 (70.9)	48 (41.0)	6.635	0.041*
15 -19 (n=183)	66 (75.0)	127 (69.4)	82 (44.8)	114 (62.3)	76 (41.5)		
**Gender**							
Male (n=137)	49 (35.8)	104 (75.9)	60 (43.8)	92 (67.2)	57 (41.6)	0.967	0.617
Female (n=163)	74 (45.4)	113 (69.3)	62 (38.0)	105 (64.4)	67 (41.1)		
**Class level**							
JSS 1-3 (n=130)	52 (40.0)	100 (76.9)	54 (41.5)	88 (67.7)	53 (40.8)	2.790	0.248
SSS 1-3 (n=170)	71 (41.8)	117 (68.8)	68 (40.0)	109 (64.1)	71 (41.8)		
**Location**							
Urban (n=181)	84 (46.4)	136 (75.1)	70 (38.7)	115 (63.5)	77 (42.5)	0.965	0.617
Rural (=119)	39 (32.8)	81 (68.1)	52 (43.7)	82 (68.9)	47 (39.5)		

* Significant at p < .05; F = frequencies; JSS= junior secondary school; SSS= senior secondary school

## Discussion

In this study, we examined secondary school students´ knowledge about Mpox in Enugu state, Nigeria. The knowledge of most students that Mpox is transmitted through contact with body fluids and that fever is associated with Mpox (Table 2) is highly commendable. This knowledge should be sustained for a healthy community. This could be because of students´ experiences in teaching and learning in related subjects such as basic sciences, health and physical education, and social studies in schools and through different media outlets. This finding agrees with [33], where 62.2% correctly identified high fever as a sign of Mpox in Nepal. However, the finding that less than one-half (40.0%) of participants know that Mpox can be transmitted from human to human, especially sexual transmission, is not encouraging. Studies have confirmed sexual transmission of Mpox disease [21,22]. Therefore, public enlightenment in wards, schools, communities, and health settings should be a priority for various stakeholders in health for Mpox-wide publicity. A little above one-half of students know that Mpox is caused by a virus (50.3%) and can be transmitted through blood transfusion (54.7%).

Our study also revealed that 41% of students possessed a good level of knowledge of causes of Mpox, with a mean knowledge score of 63.80%; 72.3% possessed a good level of knowledge of signs and symptoms; only 40.7% had a good level of knowledge of transmission, while 65.7% had a good level of knowledge of prevention. Overall, only 41.3% had a good level of knowledge of Mpox disease. Oyebade et al [34] reported similar findings in South West Nigeria, where only 37.8% had good knowledge of Mpox. Also, Al-Mustapha et al [35] reported that 58.7% of Nigerians have good knowledge of Mpox. However, this finding disagrees with that of the Nepal study [33], which reported that 72.3% had good knowledge of Mpox. These variations in knowledge could be a result of study settings and policy implementation issues. Knowledge was better in signs and symptoms and preventive practices of Mpox. This could be because the signs and symptoms of Mpox are similar to those of most other infectious diseases, and the preventive measures are similar to those of the recent global COVID-19 outbreak. A report claims that preventive measures may be hampered by a lack of Mpox understanding, especially among the general public [36]. Preventive practices of Mpox are of two prongs: animal to human, and human to human. These two should be well addressed to expose students to the disease and preventive practices to avoid further outbreaks. Less than one-half of students possessed a good level of knowledge of causes and modes of transmission. It is important to note that knowledge of the causes of Mpox is important to avoid the sickness and to help in health promotion and prevention programmes. These findings have implications for health education. The main knowledge gaps in another study in Nigeria were in incubation, signs and symptoms, mode of transmission, and prevention [35]. It implies that more work is needed to improve students´ knowledge of the causes and transmission of Mpox. This becomes very necessary if the disease is to be curbed in recent times.

Our study revealed that only the age of the students was significantly associated with good knowledge of Mpox (p < .05) (Table 4). A higher proportion of older students possessed good knowledge than younger students. This could be because age is associated with better life experiences and exposure to information. Other variables studied, such as gender, class level, and location, were, however, not significantly associated with good knowledge of the disease. No sociodemographic characteristic was associated with Mpox knowledge in Nepal [33].

Limitations for the study: like every other quantitative research, limitations include that the use of a self-reported questionnaire may not be able to assess the full extent of knowledge of Mpox disease. The respondents might be biased while responding to the questionnaire items, which might lead to the giving of false information while filling out the questionnaire. A mixed-method approach would give more detailed information on the knowledge of Mpox among students, where findings from the qualitative data could be used to corroborate the findings from the quantitative data. However, the study provided an insight into the knowledge of secondary school students about Mpox, which can be used by policy planners for an effective training programme on Mpox for educators, students, and community members in the study area.

## Conclusion

Students in secondary schools in Enugu state, Nigeria, had an average level of knowledge of Mpox disease. We recommend that Mpox education and awareness creation should go beyond school settings. It should also be extended to communities so that parents, community leaders, and other stakeholders are aware of the disease and can disseminate the information to their subjects to reduce or eliminate Mpox.

### What this study adds

- Mpox is a viral disease transmitted by monkeys and other animals to humans as well as from human to human. It was attributed to African countries alone, but became a public health emergency of continental security - PHECSl. Its continuous resurgence led to naming it a disease of public health emergency of international concern - PHEIC;
- Signs and symptoms of Mpox are almost the same as those of other infectious diseases. Thus, the need to expose the disease to the population through appropriate channels for its prevention, according to literature, vaccination was rare, especially in low-income countries such as ours;
- The risk factors for Mpox include interaction with animals, HIV and other sexually transmitted diseases, multiple sexual partners, and men who have sex with men..

### What is known about this topic

- Secondary school students in schools in Nigeria have an average level of knowledge of Mpox;
- The study added to the literature on the study of knowledge of Mpox in schools in Nigeria;
- Secondary school students have limited knowledge about the causes and mode of transmission of Mpox disease.
